# 5-Aminolevulinic acid with ferrous iron improves early renal damage and hepatic steatosis in high fat diet-induced obese mice

**DOI:** 10.3164/jcbn.18-35

**Published:** 2018-10-02

**Authors:** Atsuko Kamiya, Takeshi Hara, Masayuki Tsuda, Emi Tsuru, Yasushi Kuroda, Urara Ota, Takashi Karashima, Hideo Fukuhara, Keiji Inoue, Masahiro Ishizuka, Motowo Nakajima, Tohru Tanaka

**Affiliations:** 1 SBI Pharmaceuticals Co. Ltd., 1-6-1 Roppongi, Minato-ku, Tokyo 106-6020, Japan; 2 Institute for Laboratory Animal Research, Kochi Medical School, Kohasu, Oko, Nankoku, Kochi 783-8505, Japan; 3 Department of Urology, Kochi Medical School, Kohasu, Oko, Nankoku, Kochi 783-8505, Japan

**Keywords:** 5-aminolevulinic acid, high fat diet, diabetic nephropathy, hepatic steatosis, heme oxygenase-1

## Abstract

5-Aminolevulinic acid, a natural amino acid, activates mitochondrial respiration and induces heme oxygenase-1 expression. Obesity and type 2 diabetes mellitus are associated with age-related mitochondrial respiration defect, oxidative stress and inflammation. The aim of this study is to investigate the effects of 5-aminolevulinic acid with sodium ferrous citrate on early renal damage and hepatic steatosis. 7-Month-old C57BL/6 mice were fed with a standard diet or high fat diet for 9 weeks, which were orally administered 300 mg/kg 5-aminolevulinic acid combined with 47 mg/kg sodium ferrous citrate (5-aminolevulinic acid/sodium ferrous citrate) or vehicle for the last 5 weeks. We observed that 5-aminolevulinic acid/sodium ferrous citrate significantly decreased body weight, fat weight, hepatic lipid deposits and improved levels of blood glucose and oral glucose tolerance test. In addition, 5-aminolevulinic acid/sodium ferrous citrate suppressed increased glomerular tuft area in high fat diet-fed mice, which was associated with increased heme oxygenase-1 protein expression. Our findings demonstrate additional evidence that 5-aminolevulinic acid/sodium ferrous citrate could improve glucose and lipid metabolism in diabetic mice. 5-Aminolevulinic acid/sodium ferrous citrate has potential application in obesity or type 2 diabetes mellitus-associated disease such as diabetic nephropathy and nonalcoholic fatty liver disease.

## Introduction

5-Aminolevulinic acid (ALA) is a natural amino acid contained in various foods such as vegetables, fruits and fermented liquors.^(1,2)^ ALA is synthesized from glycine and succinyl-CoA in mitochondria by ALA synthase.^(1–4)^ ALA is a common precursor of tetrapyrroles including heme, vitamin B_12_, and chlorophyll.^(2,5)^ Especially, heme is essential for biological activity in a whole body to be a prosthetic group of hemeproteins: hemoglobin, myoglobin, P450, catalase peroxidase and several oxidative phosphorylation (OXPHOS) proteins such as cytochrome c, complex III, cytochrome *c* oxidase (COX IV). Several studies have showed that the administration of ALA activates mitochondrial respiratory function by increasing the protein expression and/or activity of COX IV and ATP production through the induction of heme production.^(6)^ The increased intracellular levels of heme induce the expression of heme oxygenase-1 (HO-1).^(7–10)^ HO-1 is one of stress inducible proteins that catalyzes heme to biliverdin, carbon monoxide, and free iron.^(11)^ Subsequently, biliverdin is converted by biliverdin reductase to bilirubin, which acts as antioxidant.^(1)^ High expression of HO-1 protect against cell injuries caused by oxidative stress in various tissues.^(9,10,12)^ Several studies demonstrated that the combination of ALA and sodium ferrous citrate (SFC) induces HO-1 expression in cultured cells of human and mice,^(7,12,13)^ or various tissues in rodents.^(8–10,14,15)^ Furthermore, this enhanced HO-1 expression protects against tissue injuries in rats under various stress conditions, such as cisplatin-induced nephrotoxicity,^(10)^ hydrogen peroxide-induced cardiomyocyte hypertrophy^(12)^ and ischemia-reperfusion induced renal injury.^(15)^

Obesity increases risks of developing abnormal glucose intolerance, hypertension, hyperlipidemia, and nonalcoholic fatty liver disease (NAFLD).^(16–18)^ Moreover, obesity is a risk factor for type 2 diabetes mellitus (T2DM) and cardiovascular disease.^(19)^ Chronic hyperglycemia condition gradually cause tissue damages including kidney, retina, and liver, resulting in diabetic complications.^(20–23)^ Two-third of type 2 diabetes patients have NAFLD.^(24)^

The administration of ALA/SFC reduced blood glucose levels and improved glucose tolerance in diabetic rodents by enhancing mitochondrial function.^(8,25–27)^ In prediabetic human subjects, the oral administration of ALA/SFC improves also glucose intolerance.^(28–30)^ Thus, we hypothesized that the administration of ALA/SFC suppressed the progression of T2DM and improved complications caused by chronic hyperglycemia and obesity through anti-oxidative effect. Identically to the preventive effects of ALA/SFC in cisplatin-induced nephrotoxicity, ALA/SFC might prevent renal damage caused by chronic hyperglycemia. ALA/SFC also reduces adiposity in diet-induced obesity (DIO) mice and 3T3-L1 adipocyte.^(25,26,31)^ Therefore, the administration of ALA/SFC has a possibility of the prevention of NAFLD.

In the present study, to clarify this hypothesis, we investigated the effects of ALA/SFC on obesity or T2DM-related diseases in kidney and liver, especially in their early stages, in C57BL/6J obese mice fed high fat diet (HFD) at middle age.

## Materials and Methods

### Animals and experiments

28-week-old male C57BL/6J mice (CLEA Japan, Tokyo, Japan) were fed with a standard diet (SD) (10% kcal fat, D12450B, Research diet, New Brunswick, NJ) or HFD (60% kcal fat, D12492, Research diet) for 9 weeks. Mice were maintained on 12 h light/12 h dark cycle in a conventional room with ad libitum feeding and drinking. The mice were fed with SD/HFD for 4 weeks (pre-feeding) and divided into three groups. Group I was a lean group fed with SD and orally administrated saline for 5 weeks after pre-feeding (*n* = 7). Group II was a metabolic group fed with HFD and orally administrated saline for 5 weeks after pre-feeding (*n* = 12). Group III was a metabolic group fed with HFD and orally administrated 300 mg/kg ALA hydrochloride and 47.1 mg/kg SFC (HFD + ALA/SFC) for 5 weeks after pre-feeding (*n* = 11). ALA was obtained from Cosmo Oil Co., Ltd. (Tokyo, Japan). SFC was purchased from Komatsuya Corporation (Osaka, Japan). Mice were performed oral glucose tolerance test (OGTT) at 36 weeks of age, in 4 weeks after oral administrations. After one week of OGTT, all mice were sacrificed under anesthesia for biochemical and histological analyses. Mice were weighed weekly. Food intakes were estimated as the difference of weights between the offered and the remnant amount of food twice per week. All mice protocols were approved by the Institutional Animal Care and Use Committee at the University of Kochi and experiments were conducted in accordance with institutional guidelines.

### Oral glucose tolerance test (OGTT)

Mice were fasted for 5 h (8:00–13:00) and OGTT were performed. Blood samples were collected from the tail vein at 0, 15, 30, 60 and 120 min after oral administration of glucose (2 g glucose/10 ml/kg). Plasma glucose level was measured by Stat Strip XP (NIPRO, Osaka, Japan).

### Measurement of biochemical parameters

Blood samples were taken from axillary artery of all mice under anesthesia at around 9:00 am on the measurement days. Plasma was prepared and the concentrations of glucose, total cholesterol (TCHO), triglyceride (TG) (Wako Pure Chemical Industries, Osaka, Japan), insulin (Morinaga Institute of Biological Science, Kanagawa, Japan), and leptin (Morinaga Institute of Biological Science, Kanagawa, Japan) were measured.

### Histological analysis of liver

Liver tissue were fixed in 10% formaldehyde and gradually replaced with sucrose. The tissues were embedded in OCT compound (Sakura Finetek Japan, Tokyo, Japan) and frozen sections were prepared. After washed with PBS, slides were placed in 60% isopropyl alcohol for 1 min. Slides were then incubated in Oil Red O solution (Sigma, St. Louis, MO) for 15 min. After replaced in 60% isopropyl alcohol for 1 min, slides were rinsed with PBS, mounted with aqueous mounting media, and cover slipped.

### Histological analysis of kidney

Kidney was fixed in 10% formaldehyde and embedded in paraffin. Tissue sections were stained with hematoxylin-eosin (H&E) and Periodic acid-Schiff stain (PAS). PAS staining was performed following the previously reported procedure.^(32)^ From each mouse, 6 glomeruli cuts were supplied for the analysis of glomerular area and diameter.

### Western blotting analysis of kidney

The detection of HO-1 protein was carried out following the previous report.^(8)^ Kidney was lysed in RIPA buffer (Wako Pure Chemical Industries, Osaka, Japan) containing 1% Halt Protease Inhibitor Cocktail. This lysate was fractionated by 4–15% gradient SDS-PAGE, and electrotransferred onto Trans-Blot Turbo Mini PVDF membranes (Bio-Rad Laboratories, Hercules, CA). The membranes were incubated with primary antibodies: anti-HO-1 antibody (kindly provided from Dr. Shigeru Taketani, Department of Biotechnology, Kyoto Institute of Technology), anti-GAPDH antibody (Enogene Biotech, New York, NY). Following incubation with HRP-linked anti-rabbit IgG antibody (GE healthcare, Princeton, NJ), HO-1 and GAPDH were detected by using Immuno Star LD (292-69903, Wako Pure Chemical Industries, Osaka, Japan) and quantified by ChemiDoc MP system (Bio-Rad Laboratories, Hercules, CA).

### Statistical analysis of data

The results were expressed as means ± SEM. Statistical significance was determined the using one-way analysis of variance (ANOVA) followed by unpaired two-tailed Student’s *t* test. *P*<0.05 was considered statistically significant.

## Results

### The administration of ALA/SFC reduces body weight and weight of white adipose tissues in HFD-induced obese mice

**Fig. 1:**
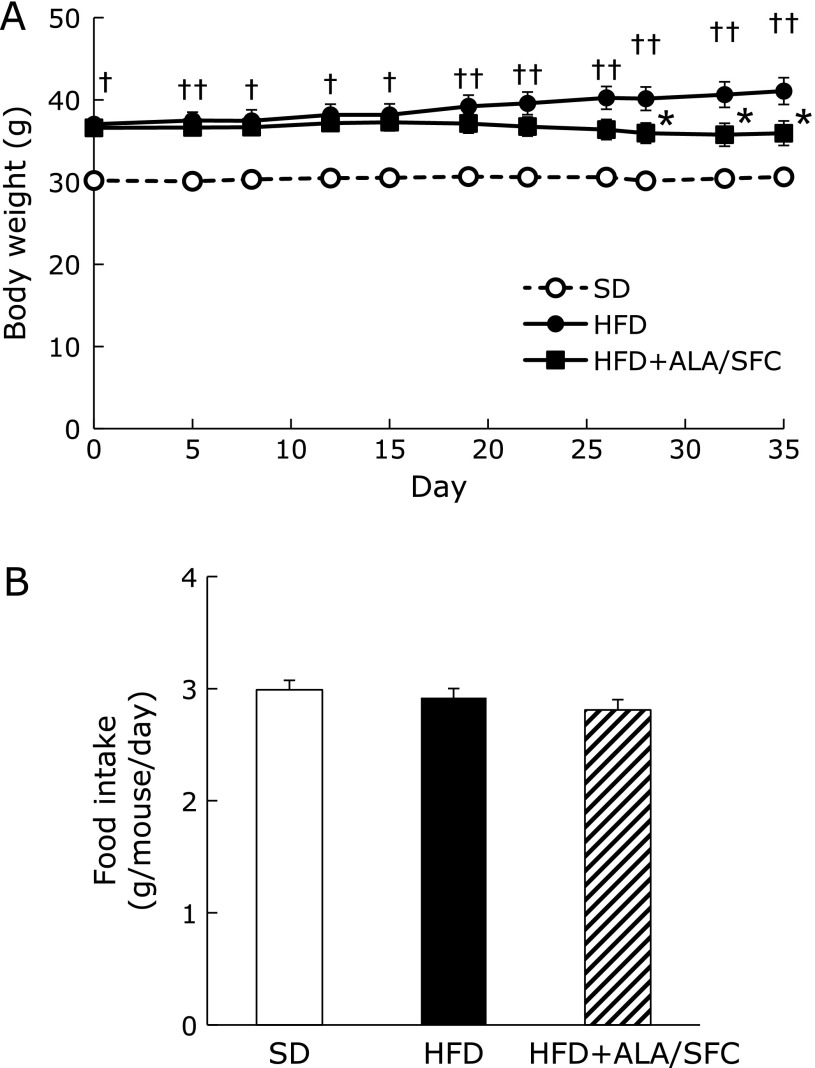
Body weights and food intakes in HFD-induced obese mice. (A) Body weights after administration of ALA. (B) Food intakes. Values are expressed as mean ± SEM. SD, standard diet group (*n* = 7); HFD, high fat diet group (*n* = 12); HFD + ALA/SFC, high fat diet with administration of ALA and SFC group (*n* = 11) (^†^*p*<0.01, ^††^*p*<0.001 vs SD group; *p<0.05 vs HFD group).

We previously reported ALA/SFC reduces body weight, weight of white adipose tissues and plasma glucose levels in DIO mice.^(25)^ First, using the middle-aged mice, we confirmed these effects on lipid and glucose metabolism. Next, we investigated the effect of ALA/SFC on kidney and liver injuries, which especially occur at early stage of obesity or T2DM-associated disease. The body weight of middle-aged HFD-fed mice administered with ALA/SFC (HFD + ALA/SFC group) for 5 weeks were significantly decreased compared with those of the obese mice administered only vehicle (HFD group) (Fig. 1A). No significant difference in food intake was observed between HFD and HFD + ALA/SFC groups (Fig. 1B). Next, we measured the weights of retroperitoneal, perirenal, epididymal and mesenteric fat in HFD + ALA/SFC group (Table 1). These fat weights in HFD + ALA/SFC group were significantly decreased as compared to those in HFD group. ALA/SFC reduced weight of body and adipose tissues in middle-aged mice fed with HFD for 5 weeks, and the effects of ALA/SFC on body and fat weight are consistent with our previous results.^(25)^

We also examined weights of kidney, liver, and gastrocnemius muscle (Table 1). The weights of kidney and gastrocnemius muscle in HFD + ALA/SFC group were significantly increased as compared to those in HFD group. The kidney weight of HFD + ALA/SFC was the same as that in SD group (Table 1), while the difference of the weight of liver in HFD + ALA/SFC group and that in HFD group was not statistically significant.

### The administration of ALA/SFC reduces increased plasma glucose levels and improves glucose intolerance in HFD-induced obese mice

We investigated the plasma glucose levels in middle-aged HFD-induced obese mice under casual or fasting conditions after the administration of ALA/SFC. The casual glucose levels in HFD + ALA/SFC group at 5 weeks after ALA/SFC administration were significantly decreased as compared with those in HFD group (Table 2).

**Fig. 2:**
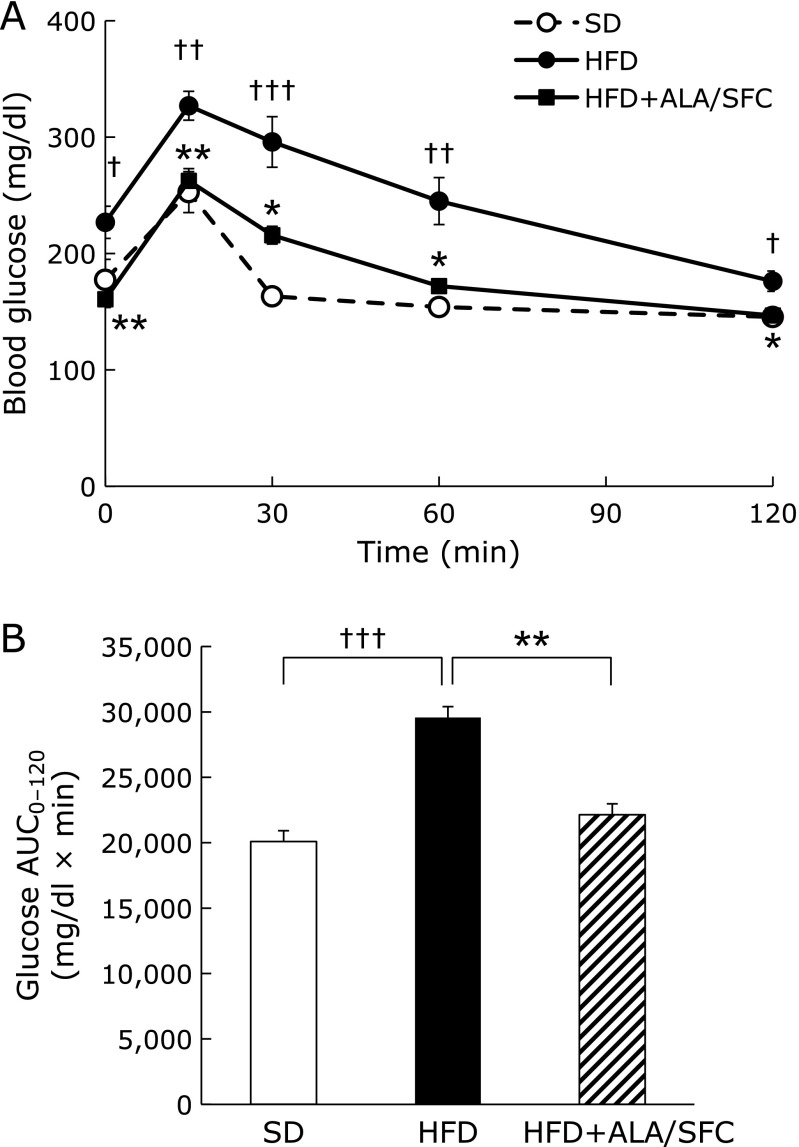
Oral glucose tolerance test (OGTT) in HFD-induced obese mice. (A) OGTT. (B) Quantification of area under the curve (AUC) from OGTT. Values are expressed as mean ± SEM. SD, standard diet group (*n* = 7); HFD, high fat diet group (*n* = 12); HFD + ALA/SFC, high fat diet with administration of ALA and SFC group (*n* = 11) (^†^*p*<0.05, ^††^*p*<0.01, ^†††^*p*<0.001 vs SD group; *p<0.01, **p<0.001 vs HFD group).

We performed glucose tolerance test (OGTT). Blood glucose levels at all measured time in HFD + ALA/SFC group were significantly lower than those in HFD group (Fig. 2A). The area under curves (AUC) in HFD + ALA/SFC group was significantly decreased as compared to those in HFD group (Fig. 2B). These results suggest that ALA/SFC reduces increased plasma glucose levels and improves glucose intolerance in HFD-induced obese mice. Those are consistent with our previous results.^(25)^

### The administration of ALA/SFC reduces casual blood glucose, insulin, leptin levels in HFD-induced obese mice

We also examined blood biochemical parameters (Table 2). The plasma levels of TCHO and TG in HFD + ALA/SFC group were not significantly different from those in HFD group. The plasma levels of TCHO in HFD + ALA/SFC group were decreased slightly, but the difference was not significant. The differences of plasma levels of TG between SD, HFD and HFD + ALA/SFC groups were not significant. The plasma levels of insulin and leptin in HFD + ALA/SFC group were decreased significantly as compared with those in HFD group.

### The administration of ALA/SFC prevents early renal damage of diabetic nephropathy in HFD-induced obese mice

**Fig. 3:**
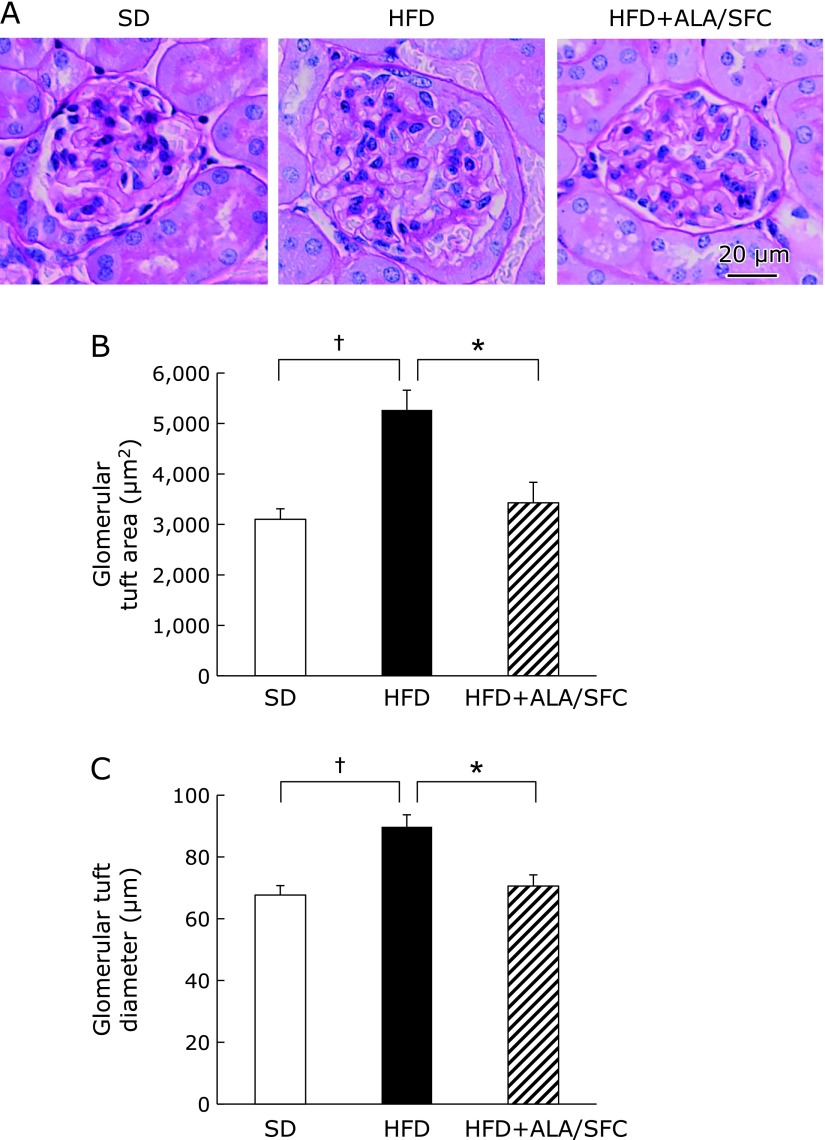
Histology of kidneys in HFD-induced obese mice. (A) Periodic acid-Schiff (PAS)-stained kidney sections in high fat diet-induced obese mice. Scale bar means 20 µm. (B) Glomerular tuft area. (C) Glomerular diameter. Values are expressed as mean ± SEM. SD, standard diet group (*n* = 7); HFD, high fat diet group (*n* = 7); HFD + ALA/SFC, high fat diet with administration of ALA and SFC group (*n* = 7) (^†^*p*<0.001 vs SD group; *p<0.01 vs HFD group).

Renal hypertrophy is associated with early renal damage in diabetic nephropathy.^(33)^ We found renal hypertrophy in the HFD group (Fig. 3A). The renal hypertrophy in HFD + ALA/SFC group was reduced as compared with that in HFD group (Fig. 3A). We performed histological analysis of glomerular tuft in kidney in HFD + ALA/SFC mice. The glomerular tuft area and its diameter in HFD + ALA/SFC group were decreased as compared to those in HFD group (Fig. 3B and C). The glomerular tuft area and its diameter in HFD + ALA/SFC group were almost similar to those in SD group. We did not find glomerulosclerosis and interstitial fibrosis in the kidney of HFD group. These data suggest that ALA/SFC prevents early renal damage of diabetic nephropathy in HFD-induced obese mice.

### The administration of ALA/SFC induces HO-1 protein expression in kidney of HFD-induced obese mice

**Fig. 4:**
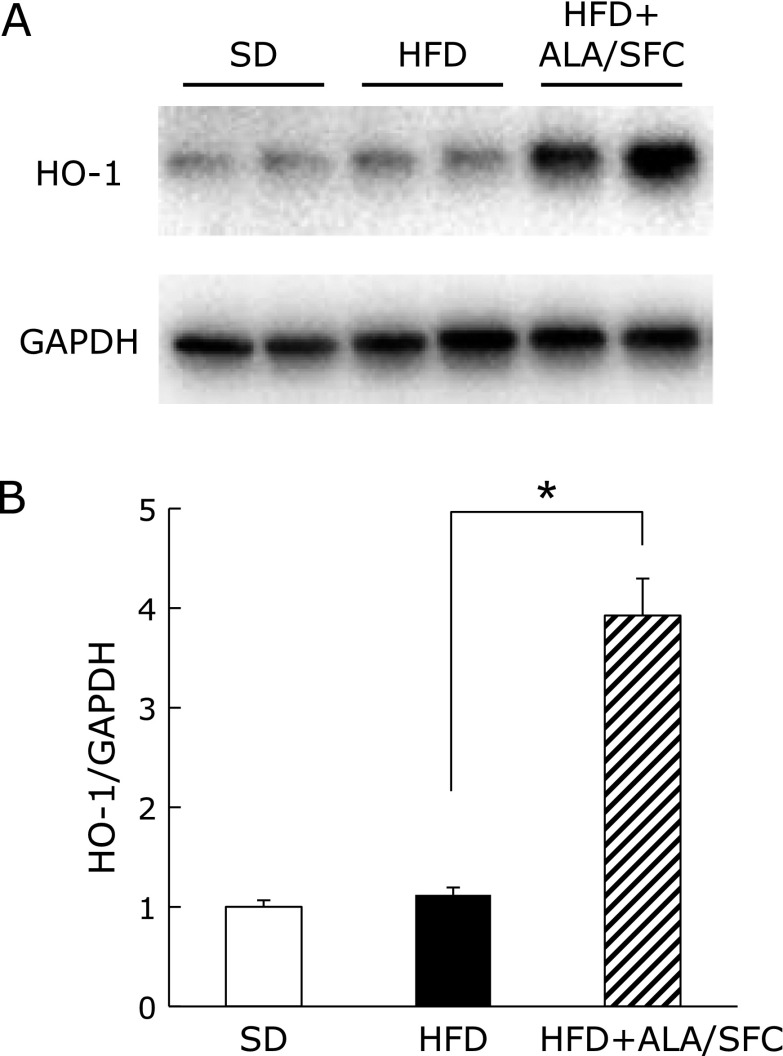
HO-1 protein expression in kidney of HFD-induced obese mice. (A) Western blot of HO-1 and GAPDH (B) Quantitative levels of HO-1/GAPDH protein expressions. Values are expressed as mean ± SEM. *n* = 7. SD, standard diet group; HFD, high fat diet group; HFD + ALA/SFC, high fat diet with administration of ALA and SFC group (*p<0.001 vs HFD group).

 Increased HO-1 expression in kidney under oxidative stress is reported to be associated with the protection against cell injury.^(34)^ Thus, we examined HO-1 expression in the kidney of HFD-induced obese mice. The HO-1 expression in kidney of HFD + ALA/SFC group was higher than that in HFD group (Fig. 4A and B), while HO-1 expressions were low in SD and HFD groups. These data suggest that ALA/SFC protect from cell injury caused by oxidative stress through the induction of HO-1 protein expression in kidney of HFD-induced obese mice.

### The administration of ALA/SFC improves slightly hepatic steatosis in HFD-induced obese mice

**Fig. 5:**
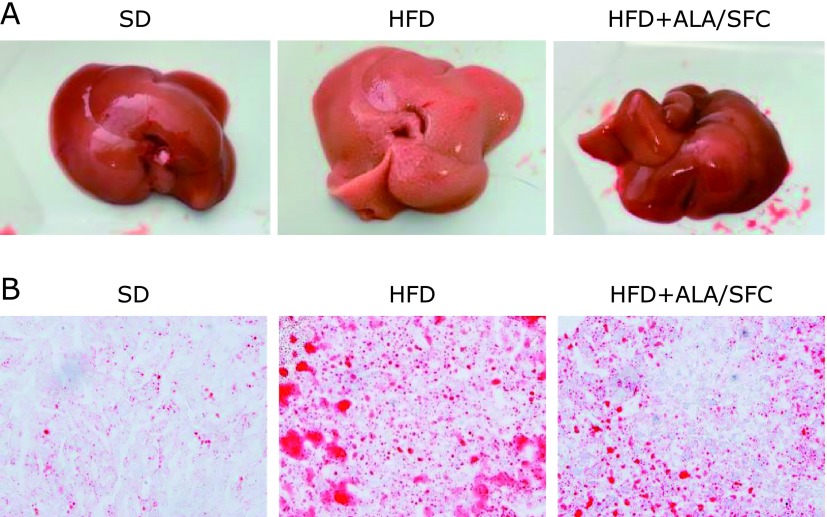
The effect of ALA/SFC on hepatic steatosis in HFD-induced obese mice. (A) Liver dissections. (B) Oil red stained liver sections. Original magnification ×200. SD, standard diet group; HFD, high fat diet group; HFD + ALA/SFC, high fat diet with administration of ALA and SFC group.

We found that the administration of ALA/SFC decreased weights of subcutaneous adipose tissue. We also investigated the effect of ALA/SFC on hepatic steatosis in HFD-induced obese mice. Livers in HFD group were whitish, which seemed to be hepatic steatosis (Fig. 5A). In addition, large lipid droplets in livers in HFD group were observed (Fig. 5B). In contrast, the livers in HFD + ALA/SFC group were red like those in SD group, implying that ALA/SFC improved the hepatic steatosis (Fig. 5A). Sizes of lipid droplets in HFD + ALA/SFC group were reduced as compared with those in HFD group (Fig. 5B). These data suggest that ALA/SFC might suppress hepatic steatosis in HFD-induced obese mice.

## Discussion

In the present study, we examined whether the administration of ALA/SFC prevents the progression of obesity or T2DM-associated diseases in HFD fed middle-aged mice. We confirmed that the administration of ALA/SFC reduced plasma glucose levels under casual and fasting conditions, and improved glucose intolerance even in HFD-induced middle-aged obese mice. ALA/SFC also reduced body weight in HFD fed mice, probably resulting from reduction of weight of subcutaneous adipose tissues. These results are compatible with our previous findings that the administration of ALA/SFC improves obesity and diabetes by activating glucose and lipid metabolism.^(8,25)^

Mitochondrial dysfunction has been reported to be associated with chronic hyperglycemia.^(35–38)^ Previous reports suggest that ALA/SFC enhances mitochondrial function through activation of electron transport chain and increases aerobic glycolysis and lipolysis metabolism, resulting in the improvements of chronic hyperglycemia and obesity. ALA/SFC also elevates level of total oxygen consumption in rats accompanied with higher expression of uncoupling protein 1 (UCP-1) in brown adipocyte tissues.^(39)^ UCP-1 is a thermogenesis protein in the inner mitochondrial membrane that dissipates energy to produce heat instead of ATP.^(40)^

Leptin is one of adipokines to decrease appetite and its level is parallel to body fat mass.^(41)^ Thus, the lower level of leptin in HFD + ALA/SFC than that of HFD indicates reduction of subcutaneous adipose weight. Further, we also found the decreased level of insulin in HFD + ALA/SFC group. Insulin secretion is regulated by glucose. Decrease of glucose level by ALA/SFC reduces insulin level indirectly.

Furthermore, we newly found that ALA/SFC prevented the hypertrophy of glomerular tuft in kidney in HFD-induced obese mice of middle age and reduced lipid droplets sizes in their livers. As described above, the improvement of chronic hyperglycemia and obesity by ALA/SFC might prevent kidney and liver against cellular damages. ALA/SFC is reported to prevent ROS production and eliminate ROS by the induction of HO-1.^(14)^ Our experiments clearly showed ALA/SFC induced HO-1 production in kidney. The chronic hyperglycemia and obesity were known to induce intracellular oxidative stress, resulting in cellular damages of various tissues such as kidney and vascular system.^(21,42–44)^ Therefore, the elimination of ROS by ALA/SFC, besides reducing glucose and lipid levels, is important for suppressing the progression of kidney damages. Several studies have revealed that antioxidants prevent the progression of diabetic nephropathy in rodents.^(45,46)^ HO-1 plays an important role as an antioxidant to prevent progression of diabetic nephropathy in mice.^(34,47)^ Furthermore, the administration of ALA/SFC protects kidney against cisplatin-induced nephrotoxicity in rats.^(10)^ ALA/SFC might prevent renal damage in diabetic nephropathy through the induction of HO-1 expression. Further studies are required for exploring underlying mechanisms to prevent diabetic nephropathy by ALA/SFC using other model mice, such as db/db or streptozotocin mice.

The large lipid droplets in hepatocytes are the hallmark of steatosis.^(48)^ We found that ALA/SFC apparently reduced the size and number of lipid droplets in the liver as compared to those in HFD group. ALA/SFC is reported to reduce lipid content in 3T3-L1 adipocyte,^(25)^ which agrees with our study results. These data suggest that ALA/SFC might prevent lipid accumulation and protect against hepatic steatosis and NAFLD. Further studies are required for elucidating the mechanism underlying lipid deposit in liver and hepatocyte treated with ALA/SFC.

In summary, we found that ALA/SFC reduced plasma glucose level, body weight, subcutaneous adipose tissue weight, hepatic lipid deposit, and improved glucose intolerance and glomerulus hypertrophy in HFD-induced obese mice. ALA/SFC induced HO-1 expression in kidneys of HFD-fed mice. Our findings suggest that ALA/SFC could prevent the progression of obesity or T2DM-associated diseases such as diabetic nephropathy and NAFLD through enhancing mitochondrial activity and inducing HO-1 as an antioxidant. Thus, ALA/SFC may become a therapeutic agent in metabolic syndrome, T2DM and T2DM-associated diseases.

## Figures and Tables

**Table 1:** Organ weights per body weight (mg/g bw) in high fat diet-induced obese mice

Group	Liver	Kidney	Retroperitoneal and perirenal fat	Epididymal fat	Mesenteric fat	Gastrocnemius muscle
SD	44.3 ± 2.8	10.9 ± 0.56	10.7 ± 1.06	21.9 ± 1.89	12.3 ± 0.70	5.6 ± 0.10
HFD	39.0 ± 2.5	8.6 ± 0.29^†^	31.2 ± 1.77^†^	53.1 ± 3.46^†^	25.9 ± 2.72^†^	4.4 ± 0.13^†^
HFD + ALA/SFC	43.5 ± 0.9	10.2 ± 0.48**	19.2 ± 2.39***	42.3 ± 5.02*	16.8 ± 1.97**	4.8 ± 0.19*

Values are expressed as mean ± SEM. *n* = 7–12. SD, standard diet group; HFD, high fat diet group; HFD + ALA/SFC, high fat diet with the administration of ALA and SFC group (^†^*p*<0.001 vs SD group; *p<0.05, **p<0.01, ***p<0.001 vs HFD group).

**Table 2:** Blood biochemistry in high fat diet-induced obese mice

	Glucose (mg/dl)	TCHO (mg/dl)	TG (mg/dl)	Insulin (ng/ml)	Leptin (ng/ml)
SD	183 ± 15.7	84 ± 10.4	72 ± 8.3	1.02 ± 0.186	7.29 ± 1.02
HFD	186 ± 10.8	140 ± 9.0^†^	80 ± 6.4	4.37 ± 1.10^†^	46.9 ± 6.71^†^
HFD + ALA/SFC	155 ± 5.9*	119 ± 9.1	91 ± 10.8	1.49 ± 0.46*	16.5 ± 2.98**

Values are expressed as mean ± SEM. Blood samples were collected at around 9:00 am on the measurement days. *n* = 7–12. TCHO, total cholesterol; TG, triglyceride; SD, standard diet group; HFD, high fat diet group; HFD + ALA/SFC, high fat diet with the administration of ALA and SFC group (^†^*p*<0.001 vs SD group; *p<0.05, **p<0.001 vs HFD group).

## Abbreviations

ALA: 5-aminolevulinic acid
AUC: area under the curve
COX IV: cytochrome *c* oxidase
HFD: high fat diet
HO-1: heme oxygenase-1
NAFLD: nonalcoholic fatty liver disease
OGTT: oral glucose tolerance test
OXPHOS: oxidative phosphorylation
SFC: sodium ferrous citrate
TCHO: total cholesterol
TG: triglyceride
T2DM: type 2 diabetes mellitus
